# Stable Suppression of Lactate Dehydrogenase Activity during Anoxia in the Foot Muscle of *Littorina littorea* and the Potential Role of Acetylation as a Novel Posttranslational Regulatory Mechanism

**DOI:** 10.1155/2013/461374

**Published:** 2013-10-23

**Authors:** Ali Shahriari, Neal J. Dawson, Ryan A. V. Bell, Kenneth B. Storey

**Affiliations:** ^1^Department of Basic Sciences, Faculty of Veterinary Medicine, Shahid Chamran University of Ahvaz, Ahvaz 6135743337, Iran; ^2^Institute of Biochemistry & Department of Biology, Carleton University, 1125 Colonel By Drive, Ottawa, ON, Canada K1S 5B6

## Abstract

The intertidal marine snail, *Littorina littorea*, has evolved to withstand extended bouts of oxygen deprivation brought about by changing tides or other potentially harmful environmental conditions. Survival is dependent on a strong suppression of its metabolic rate and a drastic reorganization of its cellular biochemistry in order to maintain energy balance under fixed fuel reserves. Lactate dehydrogenase (LDH) is a crucial enzyme of anaerobic metabolism as it is typically responsible for the regeneration of NAD^+^, which allows for the continued functioning of glycolysis in the absence of oxygen. This study compared the kinetic and structural characteristics of the D-lactate specific LDH (E.C. 1.1.1.28) from foot muscle of aerobic control versus 24 h anoxia-exposed *L. littorea*. Anoxic LDH displayed a near 50% decrease in *V*_max_ (pyruvate-reducing direction) as compared to control LDH. These kinetic differences suggest that there may be a stable modification and regulation of LDH during anoxia, and indeed, subsequent dot-blot analyses identified anoxic LDH as being significantly less acetylated than the corresponding control enzyme. Therefore, acetylation may be the regulatory mechanism that is responsible for the suppression of LDH activity during anoxia, which could allow for the production of alternative glycolytic end products that in turn would increase the ATP yield under fixed fuel reserves.

## 1. Introduction

 Lactate dehydrogenase catalyzes the reversible conversion of pyruvate to lactate, with the concomitant oxidation of NADH to NAD^+^. Under anaerobic conditions, LDH becomes an important enzyme due to its ability to regenerate NAD^+^ and allows for continued carbon flow through the glycolytic pathway to support anaerobic ATP synthesis [1]. This process can be especially important in those organisms that are exposed to hypoxic or anoxic conditions for extended periods of time and require energy balance to be maintained solely through the functioning of glycolysis.

*Littorina littorea *are marine molluscs that are native to the intertidal zones of the Atlantic coast of Europe (from Scandinavia to Spain) and have been introduced to the east coast of North America as well as several other locations around the world. Changing tides frequently expose these gill-breathing snails to prolonged oxygen deprivation at low tide [2]. Moreover, environmental conditions, such as high salinity, predation, or water pollutants can cause the snails to shut their shell openings, which over an extended period of time can also generate an anoxic exposure [3, 4]. In order to survive without oxygen, *L. littorea *undergo dramatic alterations to their cellular biochemistry so as to depress their metabolic rate and create a new balance between ATP-producing and ATP-utilizing processes. One of the more drastic changes occurs in marine snail fuel metabolism [5], with oxidative phosphorylation ceasing and glycolysis assuming primary responsibility for ATP produced during anoxia.

 Previous studies on some of the key glycolytic enzymes (glycogen phosphorylase, glycogen synthase, pyruvate kinase, and pyruvate dehydrogenase) within the foot muscle of *L. littorea* suggest that glycolytic rate slows during anoxia and that this metabolic depression may originate from the posttranslational modification of these enzymes during anoxia [5]. LDH, having been shown to be regulated in other numerous studies involving anoxia-tolerant animals [6, 7], may be an important part of this regulatory network in this marine invertebrate. This study looks at the physical and kinetic properties of D-lactate specific LDH (E.C. 1.1.1.28) from foot muscle of aerobic control and 24 h anoxic *L. littorea*, as well as the possible regulatory properties of this enzyme during marine snail anoxia. 

## 2. Materials and Methods

### 2.1. Animals

*L. littorea* were purchased from the Kowloon Market, Ottawa. The snails were placed in a 30 L tub of aerated artificial seawater (1000 mOsmol/L made using Instant Ocean Sea Salt; salinity confirmed with a buoyancy meter) at 9°C in an incubator with constant darkness. No food was provided but the water was changed periodically, and any dead snails were removed. Experiments to gather aerobic control and 24 h anoxia-exposed snails were commenced after 7 days of acclimation. Control snails were sampled directly from the aerated sea water and quickly dissected, and foot muscle samples were immediately frozen in liquid nitrogen. Tissues were stored at −80°C until use.

 For anoxia exposure, 25 snails were placed in each of several jars that were fitted with two gas ports in their lids. The containers were held on ice and contained a small amount of deoxygenated seawater (1 cm deep) which had been bubbled previously with N_2_ gas for 30 min before addition to the jars. The lids were tightened and sealed with parafilm, and then a gas line was connected to one of the ports, while the other port was opened to vent the gas. N_2_ gas was continuously bubbled into the water in the jars for a further 20 min. Then, the N_2_ line was removed, the ports were closed, and the containers were returned to the 9°C incubator for a 24 h anoxia exposure. For sampling of anoxic snails, a container of snails was removed from the incubator and placed on ice, and nitrogen gassing was reinstated. Snails were sampled from the jar and quickly dissected, and foot muscle samples were immediately frozen in liquid nitrogen. Tissues were stored at −80°C until use.

### 2.2. Sample Preparation and LDH Purification

 Frozen foot muscle samples were homogenized 1 : 5 w : v in ice-cold buffer A (50 mM KH_2_PO_4_, pH 6.5, 2.5 mM EDTA, 2.5 mM EGTA, 25 mM *β*-glycerophosphate, 10 mM *β*-mercaptoethanol, and 10% v : v glycerol). A few crystals of phenylmethylsulfonyl fluoride (PMSF) were added at the time of homogenization. Homogenates were centrifuged at 13, 500 ×g for 30 minutes at 4°C. Following centrifugation, samples were collected and held on ice until use.

 LDH partial purification began with the preparation of a blue agarose column that was equilibrated in buffer B (25 mM KH_2_PO_4_, pH 6.5, 1.25 mM EDTA, 1.25 mM EGTA, 12.5 mM *β*-glycerophosphate, 5 mM *β*-mercaptoethanol, and 5% v : v glycerol). Following equilibration, approximately 2 mL of crude muscle extract was placed on top of the column. The column was then washed with 50 mL of buffer B to remove any unbound proteins. A linear salt gradient of 0–2 M KCl in buffer B was then applied to the column for the elution of LDH. This sample was used for subsequent kinetic characterization of LDH.

 Additional experiments required a pure LDH sample, and therefore, the top peak activity fractions from the blue agarose column were combined and chromatographed on a hydroxyapatite column preequilibrated in buffer B. LDH was subsequently eluted from this column using a 0–4 M KCl gradient in buffer B. Peak activity fractions were then pooled and held at 4°C until use. Protein purity was determined via SDS gel electrophoresis with subsequent gel staining with Coomassie Brilliant Blue dye (details given in separate subsection below).

### 2.3. LDH Assay

 LDH activity was monitored by the production or consumption of NADH. The optimal assay conditions for LDH in the pyruvate-reducing direction were 25 mM KH_2_PO_4_, pH 6.5, 2.5 mM pyruvate, and 0.25 mM NADH. The optimal assay conditions for the D-lactate-oxidizing direction were 25 mM KH_2_PO_4_, pH 9.5, 3 mM NAD^+^, and 30 mM D-lactate. Assays were conducted using a Thermo Labsystems Multiskan spectrophotometer at 340 nm. One unit of LDH activity is defined as the amount of enzyme that produced or utilized 1 *μ*mol of NADH per minute at 25°C. 

 Inhibition constants (*I*_50_), the amount of inhibitor that reduced enzyme activity by 50%, for urea, guanidine hydrochloride (GnHCl), KCl, and NaCl were determined by assaying foot muscle LDH under optimal conditions for the pyruvate-reducing reaction with the addition of various concentrations of the above effectors. 

 Data was analyzed using the Kinetics v.3.5.1 program [8]. Protein concentrations were determined using the Coomassie blue dye-binding method with the BioRad prepared reagent and bovine serum albumin as the relative standard.

### 2.4. Arrhenius Plots

 Maximal LDH activity was determined at 5°C increments starting from 5°C and ending at 30°C. The reaction temperature was altered by placing the Thermo Labsystems Multiskan spectrophotometer into a precision low temperature incubator 815 set to the desired temperature. Microplates filled with assay mixture (but without enzyme) were equilibrated in the same incubator for several minutes until the desired temperature was reached (as measured by a telethermometer). Plates were then placed into the spectrophotometer and reactions were initiated by the addition of enzyme. Arrhenius plots were constructed from these experiments and the activation energy (*E*_*a*_) was calculated.

### 2.5. Differential Scanning Fluorimetry

 Differential scanning fluorimetry (DSF) is a high throughput method that monitors the thermal unfolding of proteins in the presence of a fluorescent dye [9]. Purified control and 24 h anoxic LDH were aliquoted to a concentration of approximately 0.1 *μ*g/*μ*L/well into the wells of a 96-well, thin-walled PCR plate along with the dye SYPRO Orange (40X final concentration) to a total volume of 20 *μ*L. PCR plates were then sealed with sealing tape and placed into a BioRad iCycler5 PCR instrument. SYPRO Orange (Invitrogen) fluorescence was monitored as described by Biggar et al. [10]. Briefly, SYPRO Orange was excited through the transmission of light through the FAM filter (485 ± 30 nm), with the subsequent emission of light through the ROX filter (625 ± 30 nm). Measurements were taken every 30 s at 1°C increments from 25°C to 97°C. Subsequent analysis of the fluorescent data using OriginPro 8.5 and the Boltzmann distribution curve yielded the midpoint temperature of the protein unfolding transition, known as the protein melting temperature (*T*_*m*_), for control and anoxic foot muscle LDH.

### 2.6. ProQ Diamond Phosphoprotein Staining

Partially purified control and 24 h anoxic LDH samples were mixed 1 : 1 (v : v) with SDS loading buffer (100 mM Tris buffer, pH 6.8, 4% w : v SDS, 20% v : v glycerol, 0.2% w : v bromophenol blue, and 10% v : v 2-mercaptoethanol) and boiled for 5 minutes, cooled on ice, and frozen at −20°C until use.

SDS resolving gels (10% v/v acrylamide, 400 mMTris, pH 8.8, 0.1% w/v SDS, 0.2% w/v ammonium persulfate (APS), and 0.04% v/v TEMED) were prepared with a 5% stacking gel (5% acrylamide, 190 mMTris, pH 6.8, 0.1% w/v SDS, 0.15% w/v APS, and 0.1% v/v TEMED). Partially purified control and 24 h anoxic LDH were loaded onto these gels and separated electrophoretically in SDS-PAGE running buffer (25 mMTris-base, 190 mM glycine, and 0.1% w/v SDS) at 180 V for 45 min. A 3 *μ*L aliquot of FroggaBio Protein Ladder (Cat# PM005-0500) was added to one lane of every gel to provide molecular weight markers, and an aliquot of commercially pure *Lactobacillus leichmannii* D-LDH (Sigma) was also loaded in another lane to help confirm the location of the snail LDH subunits. Following electrophoresis, proteins were fixed in fixing solution (50% v : v methanol and 10% v : v acetic acid) overnight at 4°C. The gel was subsequently washed three times in ddH_2_0 for 10 minutes each time and then stained with ProQ diamond phosphoprotein stain (Invitrogen, Eugene, OR) for 1 h. During the staining process, and thereafter, the gel remained covered in aluminum foil to protect the light sensitive stain. After staining the gel was destained by washing twice in destaining solution (20% acetonitrile, 50 mM sodium acetate, and pH 4) for 30 minutes each time. The gel was then washed in ddH_2_0 three times for 5 minutes each time before the fluorescent signal was viewed in a ChemiGenius Bioimaging System (Syngene, Frederick, MD). The fluorescent bands were then quantified using GeneTools software. An identical gel was run in concert with the above gel, but was stained with Coomassie blue (25% w : v Coomassie Brilliant Blue R in 50% v/v methanol, 7.5% v : v acetic acid). The LDH ProQ band intensities were subsequently normalized against the corresponding Coomassie blue LDH band intensities for the same samples.

### 2.7. Dot-Blot Assessment of Posttranslational Modification

 Posttranslational modification of purified LDH (other than phosphorylation) was assessed using the dot-blotting protocol outlined by Dawson et al. [7]. Briefly, purified LDH from control and 24 h anoxic foot muscle was applied in equal amounts (approximately 5 *μ*g) to nitrocellulose membranes using a Bio-Dot microfiltration apparatus (Bio-Rad). Samples were allowed to filter through the membrane via gravity, after which the membranes were washed three times for 5 minutes each time in Tris-buffered saline containing 0.05% Triton-X (TBST). The membranes were then blocked with large molecular weight PVA (70–100 kDa) for 30 seconds, followed by three washes for 5 minutes each time in TBST. The nitrocellulose membranes were then incubated in one of the following primary antibodies (1 : 1000 v : v dilution) specific for particular posttranslational modifications:

- acetylation-pan-acetyl (C4)-R (sc-8663-R), anti-rabbit, Santa Cruz Biotechnology, Santa Cruz, CA, USA;
- ubiquitination-anti-ubiquitin (ab19247), anti-rabbit, Abcam, Cambridge, UK;
- methyl-lysine-anti-methylated lysine (SPC-158F), anti-rabbit, StressMarq Biosciences Inc., Victoria, BC, Canada;
- methyl-arginine-anti-methylated arginine (ab414), anti-mouse, Abcam, Cambridge, UK.

The membranes were incubated with primary antibody for 4 h at room temperature. This was followed by three washings of TBST for 5 minutes each time. Secondary antibody was subsequently added onto the blots for 45 minutes at room temperature. After this incubation, the blots were washed again three times in TBST for 5 minutes each time. Dots were then visualized with enhanced chemiluminescence on a ChemiGenius Bioimaging System (Syngene, Frederick, MD, USA). Dot intensities were quantified using GeneTools software and then subsequently normalized to protein content of each dot, assessed through Coomassie blue staining of the analyzed blots.

### 2.8. Statistical Analyses

 All kinetic measurements for control and 24 h anoxic foot muscle LDH were compared using Student's *t*-test (2-tailed, unequal variance, *P* < 0.05). The same statistical analysis was conducted for all experiments assessing the posttranslational modifications of LDH. 

## 3. Results and Discussion

### 3.1. LDH Purification

**Figure 1:**
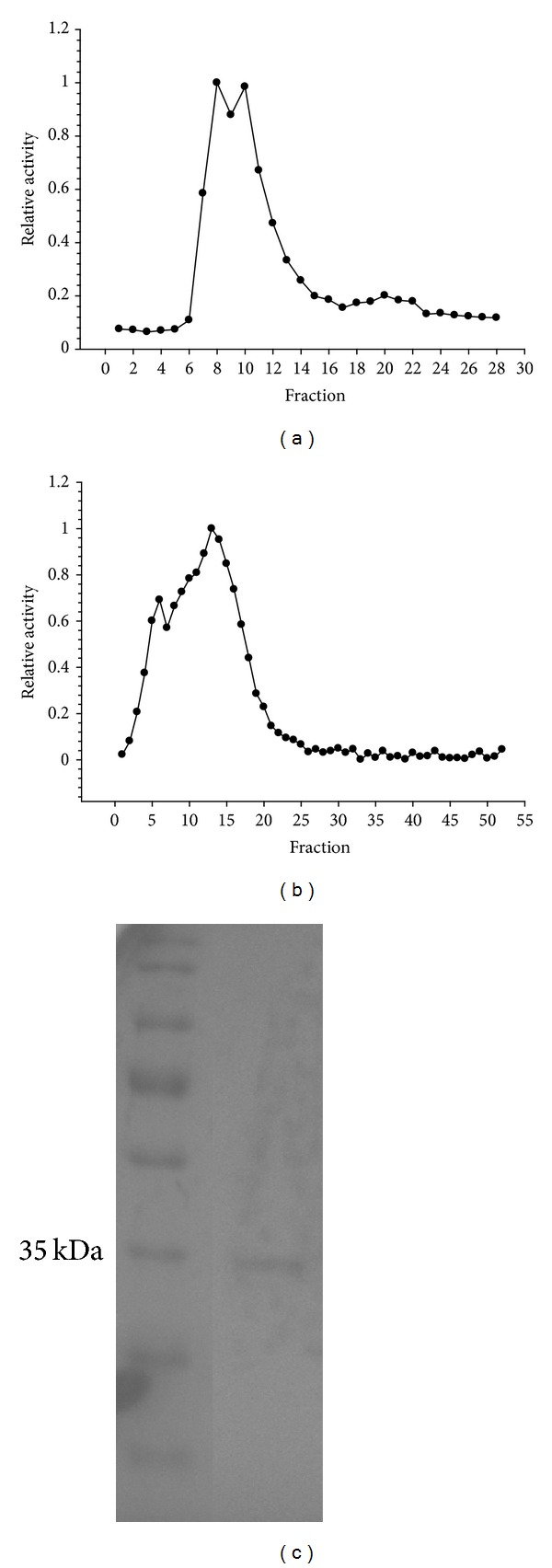
Purified control foot muscle LDH from *L. littorea*. (a) A representative blue agarose column elution profile. (b) A representative hydroxyapatite column elution profile that followed a blue agarose column. (c) Coomassie stained gel with protein ladder on left and purified control LDH on right.

 LDH was purified to electrophoretic homogeneity using blue agarose and hydroxyapatite columns (Figure 1). The resulting purified sample had a specific activity of 4.7 U/mg and an overall yield of 25% (Table 1). Other purifications of D-LDH, mainly from bacterial species, yielded specific activities that ranged from 1.6 U/mg to 1500 U/mg [11]. *L. littorea* muscle LDH specific activity was reasonably within this range.

### 3.2. LDH Kinetics

 LDH from the foot muscle of control and 24 h anoxic marine snails was partially purified prior to kinetic analysis so as to remove interfering metabolites and enzymes. Kinetic analyses indicated that control and anoxic forms of the enzyme did differ in several kinetic parameters. For instance, the *V*_max⁡_ in the pyruvate-reducing direction for anoxic LDH was approximately 50% of the control value. Furthermore, the *I*_50_ values for ATP and ADP were approximately 2-fold and 5-fold higher, respectively, for anoxic LDH as compared to the control (Table 2), while somewhat rare, inhibition of lactate dehydrogenase by ATP has been shown before in numerous bacteria [12], and as seen in this study, it is a very weak inhibitor. ADP on the other hand, does not seem to be a well-known inhibitor of either D- or L-lactate dehydrogenases but again is a weak inhibitor with *I*_50_ values being above 20 mM. It is highly unlikely that both ATP and ADP are key regulators of LDH *in vivo *as their corresponding *I*_50_ values far exceed the physiological concentrations of those metabolites [13]. Thus, the difference in LDH *V*_max⁡_ (in the pyruvate-reducing direction) is the kinetic parameter that is most significant to this animal *in vivo*. In accordance with this finding is the 16% increase in the activation energy (*E*_*a*_) for anoxic LDH as compared to the control enzyme at a pH (pH 6.5) that is typically found in fully anoxic animals (Table 2) [14–16]. These findings indicate that there may be reduced LDH activity during anoxia, and this may serve to ensure the continued processing of pyruvate by alternative enzymes to increase the ATP yield per glucose molecule oxidized. Specifically, the production of succinate and propionate increases the ATP produced per glucose oxidized from 2 to 4 and 6 molecules of ATP, respectively, [17–20]. This would be beneficial, as the marine snail must produce all of the necessary cellular energy from its fixed fuel reserves.

### 3.3. Structural Characteristics of LDH

 The kinetic differences outlined above indicate that LDH from control and anoxic conditions may exist in distinct structural states. Initial structural studies involved exposing LDH to increasing amounts of denaturants (urea and GnHCl) to determine if LDH from either control or anoxic conditions was more susceptible to inhibition. These experiments showed no significant changes in LDH *I*_50_ values for urea or GnHCl between control and anoxic conditions (Table 3). Further structural experiments were conducted using common salts, NaCl and KCl, which can affect enzyme structure by disturbing intramolecular ion-pair interactions. These studies indicated that anoxic LDH was slightly less susceptible to KCl inhibition as compared to normoxic LDH (Table 3), while NaCl affected control and anoxic LDH to the same degree. *I*_50_ values for KCl were near 1 M and, thus, are likely to have little actual significance for the enzyme *in vivo*; however, these data provide evidence that control and anoxic LDH are present in distinct structural forms.

The final structural assessment involved the thermal unfolding of purified LDH in a process termed differential scanning fluorimetry. These experiments indicated that the *T*_*m*_, the point at which 50% of the protein in an unfolded state, was significantly altered between normoxic and anoxic LDH; anoxic LDH displayed a nearly 2°C decrease in *T*_*m*_ as compared to the control condition (Table 3). Previous experiments involving turtle liver LDH identified a similar reduction in LDH *T*_*m*_ for the anoxic form of the enzyme by approximately 3°C [6]. The decrease in *T*_*m*_ observed in these two studies may signify that anoxic LDH is structurally more flexible than the corresponding control enzyme. This could allow for some level of low temperature function when the anoxic animal is exposed to frigid air temperatures that typically accompany anoxia exposure. Increased structural flexibility is a common characteristic for enzymes in animals that must function through periodic exposures to cold temperatures, as it allows increased functionality of the enzyme while sacrificing some stability (reviewed in [21]). The significance of the alteration in melting temperature can only be speculative, but nonetheless, this experiment does suggest that control and anoxic LDH exist in two different structural forms.

### 3.4. Posttranslational Modification of LDH

**Figure 2:**
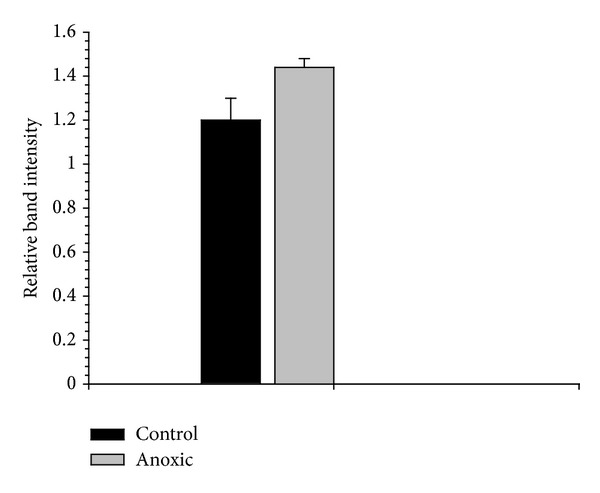
Phosphorylation state of partially purified control and 24 h anoxic foot muscle LDH as assessed through the staining of an SDS gel with ProQ Diamond phosphoprotein stain. Data are means ± SEM,  *n* = 3 independent determinations on separate enzyme samples.

 The structural dissimilarities and kinetic differences between aerobic control and 24 h anoxic LDH could be evidence of some difference in the posttranslational modification of LDH between the two conditions. One of the most common posttranslational modifications within cells is reversible protein phosphorylation. The phosphorylation state of control and anoxic LDH was assessed using ProQ Diamond phosphoprotein staining of an SDS gel containing partially purified LDH. The resulting analysis of the fluorescent band intensities indicated that there was no significant difference between control and anoxic LDH with respect to its phosphorylation state (Figure 2). Reversible phosphorylation has been shown previously to be very important in regulating LDH kinetic changes [22, 23] and has been implicated as an important regulatory mechanism in an anoxia-tolerant freshwater turtle [6, 7]. However, phosphorylation does not appear to be a crucial regulatory mechanism by which LDH activity is suppressed during anoxia in this study.

**Figure 3:**
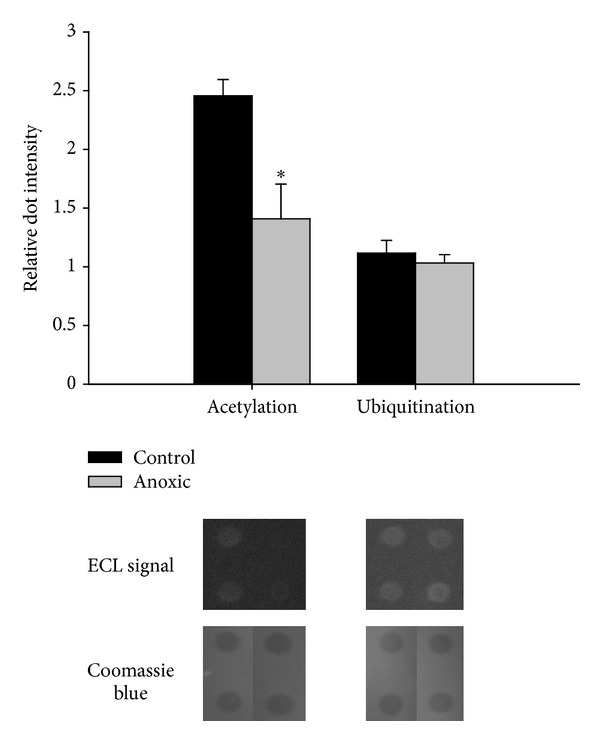
Analysis of the posttranslational acetylation and ubiquitination of control and 24 h anoxic foot muscle LDH through the use of dot blots. Chemiluminescent and Coomassie stained dots are shown below their corresponding bars as representative dots typically seen in the analysis. Data are means ± SEM, *n* = 4. *indicates a significant difference between the control and anoxic values as determined by Student's *t*-test,  *P* < 0.05.

Additional potential posttranslational modifications that could affect LDH were then investigated to determine if there was an alternate regulatory mechanism that could be responsible for suppressing LDH activity during anoxia. Dot-blot analysis of purified LDH revealed that neither control nor 24 h anoxic LDH contained any detectable methylated lysine or arginine residues (blots not shown), and thus these modifications were unlikely to be major regulators of LDH activity under anoxic conditions. Similarly, ubiquitination was detected for both control and anoxic LDH but was not significantly different between the two enzyme forms (Figure 3). Posttranslational acetylation of LDH, on the other hand, showed a distinct difference between control- and anoxia-derived enzymes; indeed, anoxic LDH showed nearly 50% less acetylation than the corresponding control enzyme (Figure 3). Reversible lysine acetylation is emerging as a major regulatory mechanism in cellular metabolism [24] and may be a key mediator of LDH activity during anoxia. Indeed, a recent study on skeletal muscle LDH from the freeze-tolerant wood frog indicated that dehydration stress brought about by freezing caused a statistically significant decrease in LDH acetylation [25]. Thus, acetylation may be emerging as a critical mediator of LDH activity in response to anoxia or other cellular stresses, and future experiments will need to be devised to alter the acetylation state of LDH to determine the metabolic consequences of this posttranslational modification.

## 4. Conclusion

 Kinetic analysis of LDH from foot muscle of control and 24 h anoxic snails revealed that oxygen deprivation may lead to a significant decrease in LDH activity. This apparent decrease in activity may be due to the stable modification of LDH due to differential LDH acetylation. The decrease in LDH activity is in accordance with the overall metabolic rate suppression observed under anoxic conditions in *L. littorea* and may serve to help alter the processing of pyruvate into alternate glycolytic end products, such as succinate, alanine, propionate, or alanopine. Further processing of pyruvate allows for increased energy production per glucose molecule oxidized and decreases the production of acidic metabolic end products, which is essential for long-term anoxia survival.

## Figures and Tables

**Table 1:** Purification scheme for foot muscle D-LDH from aerobic control *L. littorea* LDH.

Purification step	Total protein (mg)	Total activity (U)	Specific activity (U/mg)	Fold purification	% yield
Supernatant	31	3.6	0.1	—	—
Blue agarose	2.3	2.1	0.9	9	58
Hydroxyapatite	0.2	0.9	4.7	47	25

**Table 2:** Kinetic and structural analysis of partially purified control and 24 h anoxic foot muscle LDH.

	Control	24 h anoxic
Pyruvate *K*_*m*_ (mM)	0.29 ± 0.01	0.27 ± 0.01
Lactate *K*_*m*_ (mM)	25 ± 2	23.6 ± 0.8
*V*_max⁡_ (pyruvate-reducing direction; U/g wet weight)	64 ± 1	38 ± 1*
ATP *I*_50_ (mM)	13 ± 1	28 ± 3*
ADP *I*_50_ (mM)	23 ± 2	100 ± 6*
Succinate	N.E.	N.E.
Aspartate	N.E.	N.E.

*E*_*a*_ (at pH 6.5; Kcal/mol)	62.4 ± 0.5	72 ± 1*
*E*_*a*_ (at pH 7.4; Kcal/mol)	58 ± 3	64 ± 3

The data are means ± SEM, *n* ≥ 3 independent determinations on individually prepared samples. *indicates a significantly different result as compared to the control value using Student's *t*-test, *P* < 0.05. Note that the activation energy (*E*_*a*_) values for control and 24 h anoxic LDH were assessed on fully pure enzyme samples.

**Table 3:** The effects of salts, common denaturants, and temperature on control and 24 h anoxic *L. littorea *foot muscle LDH.

	Control	24 h anoxic
NaCl *I*_50_ (M)	0.79 ± 0.03	0.79 ± 0.03
KCl *I*_50_ (M)	0.97 ± 0.04	0.81 ± 0.02*
Urea *I*_50_ (M)	3.1 ± 0.1	3.1 ± 0.2
GnHCl *I*_50_ (M)	0.384 ± 0.007	0.40 ± 0.01
*T*_*m*_ (°C)	51.9 ± 0.3	50.0 ± 0.1*

The data are means ± SEM, *n* ≥ 3 independent determinations on individual enzyme preparations. *indicates that the 24 h anoxic value is significantly different from the corresponding control value using Student's *t*-test, *P* < 0.05.
